# European Health Interview Survey (EHIS) 2 – Background and study methodology

**DOI:** 10.25646/6228

**Published:** 2019-12-11

**Authors:** Birte Hintzpeter, Jonas D. Finger, Jennifer Allen, Ronny Kuhnert, Stefanie Seeling, Jürgen Thelen, Cornelia Lange

**Affiliations:** 1 Robert Koch Institute, Berlin Department of Epidemiology and Health Monitoring; 2 Formerly Robert Koch Institute, Berlin Department of Epidemiology and Health Monitoring

**Keywords:** STUDY METHODOLOGY, EHIS 2, EUROPEAN COMPARISON, EU, HEALTH MONITORING

## Abstract

The scientific assessment of health issues, the design and further development of political guidelines as well as the targeted planning of measures in the European Union (EU) require data on population health. For this reason, all EU Member States regularly collect data on the health status, provision of healthcare, health determinants and socioeconomic situation of their respective populations in the European Health Interview Survey (EHIS). Participants are at least 15 years old and live in private households. The second wave of EHIS (EHIS 2) was conducted between 2013 and 2015. For EHIS 2, each EU Member State drew a nationally representative population sample from population registers, censuses, dwelling registers or other statistical or administrative sources. Data collection modes within individual EU Member States were used, according to nationally established methods, including the use of mixed-mode surveys. Across all EU Member States, data collection took an average of eight months to complete. Member States made considerable efforts to achieve the highest possible response rates. The harmonised EHIS data collected are highly comparable and constitute an important information base for European health policy and health reporting.

## 1. Introduction

The European Union (EU) has evolved from a number of predecessor organisations. The present EU was founded on 1 November 1993 with twelve Member States. Since then, the number of Member States has steadily increased [1]. At the point of data collection for the second wave of the European Health Interview Survey (EHIS 2), the EU had 28 Member States (EU 28) and around 507 million inhabitants (in the year 2014) [2]. The present article describes the study methodology applied in EHIS 2, on which the analyses in the present issue of the Journal of Health Monitoring are based.

Current health challenges faced by the EU include not only outbreaks of disease but also longer-term developments such as urbanisation, demographic changes, food insecurity, climate change and imbalances in the provision of care within and between EU Member States [3]. Political decision-makers require reliable and up-to-date data on health. Standardised data collections based on European health indicators are of key importance to the design of national and European-level research and health policies. Moreover, data is also required for the scientific assessment of health issues and the targeted planning of specific measures. In the face of these challenges, European comparisons of health status, provision of healthcare, health determinants and socioeconomic situation play an important role. In terms of national health reporting, the EHIS results are an important data source for the comparative evaluation and classification of chronological developments.

**GEDA 2014/2015-EHIS**

**(for international comparisons)**

**Data holder:** Robert Koch Institute

**Aims:** To provide reliable information about the population’s health status, health behaviour and health care in Germany, with the possibility of a European comparison

**Method:** Questionnaires completed on paper or online

**Population:** People aged 15 years and above with permanent residency in Germany

**Sampling:** Registry office sample; randomly selected individuals from 301 communities in Germany were invited to participate

**Participants:** 24,824 people (13,568 women, 11,256 men)

**Response rate:** 27.6%

**Study period:** November 2014 - July 2015

More information in German is available at www.geda-studie.de and Lange et al. 2017 [14]

Health data are held by Eurostat [4], the Statistical Office of the European Union, the Organization for Economic Cooperation and Development (OECD) and the World Health Organization (WHO) [5, 6]. These data are published at regular intervals. The OECD Health at a Glance report [7], for example, is published every two years, while the European Health Report is published every three years [8]. The latter is published jointly by the WHO’s Regional Office for Europe and the European Commission. European health monitoring is supported by a diverse set of indicator systems, including the European Core Health Indicators (ECHI) [9], the EU social indicators and the health-related indicators from the European Sustainable Development Strategy [10]. The European Health Interview Survey (EHIS), which is described in the present article provides around one-quarter of the ECHI indicators implemented [11, 12].

All the EU Member States collect data for the EHIS on the health, provision of healthcare, health determinants and socioeconomic situation of their respective populations (Info box). EHIS is a population-based, cross-sectional survey based on the self-report of participants. Each Member State is free to decide on the survey method used and the way in which the survey is conducted. The EHIS can, for example, be conducted as a stand-alone survey, or be embedded within a national health survey, as it is the case in Germany. Both the target population (persons aged 15 and above living in private households residing in the territory of the Member State at the time of the data collection) and the sample size to be achieved by each Member State (around 195,000 participants across all EU Member States) are mandatory. The (first) voluntary survey (EHIS 1) was conducted between 2006 and 2009 [13]. Representatives from several Member States established a taskforce to develop a model questionnaire, guidelines and recommendations for translation. Wide-ranging experience in national health surveys contributed to this process. Seventeen Member States including Germany participated in EHIS 1.

Data collection for EHIS wave 2 (EHIS 2), which was legally binding for all 28 Member States (including Norway, Iceland and Turkey), was carried out between 2013 and 2015 [10, 14]. A quality report, to be completed by each participating country according to pre-defined criteria contains detailed information on their chosen methodological approach. As there is no data available from Turkey in the quality report, the current article predominantly uses data from the remaining 30 participating countries in EHIS 2 [15].

In Germany, EHIS is part of the health monitoring that takes place at the Robert Koch Institute [14]. EHIS 2 was integrated into the German Health Update (GEDA 2014/2015-EHIS). The survey was based on a two-stage cluster sample, randomly drawn from population registers. GEDA 2014/2015-EHIS was conducted between November 2014 and July 2015, using a sequential mixed-mode design with online and paper questionnaires. Lange et al. 2017 contains a detailed description of the methodology applied in GEDA 2014/2015-EHIS [14].

**Info box:**

**European Health Interview Survey (EHIS)**

The European Core Health Indicators (ECHI) were jointly developed by EU Member States and international organisations, taking into account scientific and health policy requirements. The indicators provide a framework in European health reporting for population-based health surveys and analyses, and health care provision at the European and national level. The European Health Interview Survey (EHIS) is a key element in this regard. The first EHIS wave (EHIS 1), which was not mandatory, was conducted between 2006 and 2009. 17 Member States and two non-EU countries participated in EHIS 1. Participation in the second wave of EHIS (EHIS 2), which was conducted between 2013 and 2015 in all EU Member States (as well as in Iceland, Norway and Turkey) was legally binding and is based on Commission Regulation (EU) No 141/2013 of 19 February 2013. It provides essential information about the ECHI indicators. In Germany, EHIS is carried out as part of health monitoring at the Robert Koch Institute. During the EHIS 2 survey period, the EU had 28 Member States.

Further information is available at: https://ec.europa.eu/eurostat/web/microdata/european-health-interview-survey

For participating countries (EU Member States plus Norway, Iceland and Turkey), the Eurostat website provides aggregated data (macrodata) for a diverse set of EHIS 2 health indicators [4]. Furthermore, for research purposes such as the analyses presented in this issue of the Journal of Health Monitoring, applications can be made to Eurostat in order to access anonymised data from EU Member States at participant level (microdata) [16].

## 2. Methodology

### 2.1 Development of EHIS 2

Design of the compulsory second wave of EHIS began with an intensive evaluation of EHIS 1. The experience of EHIS 1 had shown the problematic nature of individual modules from the questionnaire. Among other things, this involved sensitive questions on health-related well-being and alcohol consumption which were not consistently understood or interpreted in the different countries and cultures [17-19].

The scope of the survey instrument was also viewed critically. It was therefore decided to carefully revise the questionnaire, with the aim of using the established survey instruments to gather data with the greatest possible comparability. With this in mind, Eurostat commissioned an 18-month research project collaboration between three institutes (the Robert Koch Institute, the former Scientific Institute of Public Health - now Sciensano - in Belgium and the Estonian Institute for Health Development), starting in February 2010. The project aimed to identify the problems that had surfaced in EHIS 1, as well as develop and test question modules for mental health, alcohol consumption and physical activity [17]. The overall aim was to develop survey instruments that enable so-called ‘input harmonisation’. This means that questions should be comparable at the point of data collection (in contrast to ‘output harmonisation’ where different questions or wordings of questions are subsumed into a uniform indicator). However, full input harmonisation has its limits in a study of this size, implemented in over 28 countries, since the different survey methods used in each country can lead to varying operationalisations of questions. In addition, ensuring that people’s understanding of a question is comparable depends not only on a standardised translation but also on a shared understanding of the question’s underlying concept, which can lead to different formulations of questions.

During a three-year process that built upon the results of the research project mentioned previously, a model questionnaire for EHIS 2 was developed and finalised (chapter 2.4) with the participation of representatives from all Member States [20]. EHIS implementation is regulated by the Commission Regulation (EU) No 141/2013 of 19 February 2013 [21], which contains seven articles covering the scope, required data, reference year, reference population, reference metadata and submission of data to Eurostat. Following the accession of Croatia to the European Union, this regulation was amended by Commission Regulation (EU) No 68/2014 [22]. The implementing regulation contains the target variables of the survey questions. Use of the model questionnaire is recommended to ensure the greatest possible input harmonisation [21].

To support both the data collection in the Member States and the comparability of results, Eurostat and external experts from EU Member States developed a comprehensive manual [20]. This contains guidelines, for example on the translation process and on the sequence of questions. It also suggests a question along with response categories for each individual target variable and provides precise indications in regard to interviewers and implementation for each individual question. The Statistical Guidelines specify aspects of study design, sampling, sample size, weighting, as well as other technical details of the survey [20].

### 2.2 Study design and participants

The study population consists of the EU population living in private households [15]. People living in the overseas territories of the Netherlands, France, Ireland and the UK are exempt, as they are not part of the frame population. The EHIS 2 sample is composed of the national representative samples from the participating EU Member States. Member States used different sampling frames for drawing their national samples: population register, dwelling register and censuses, as well as other statistical sources [15].

The mandatory minimum effective national sample size under the EHIS implementation regulation was defined according to a standardised calculation method [20]. Practical, cost-related and statistical considerations are thereby taken into account. The specified sample size aims to ensure that for each EU country, a prevalence of 8% can be estimated with less than 1% point error (i.e. with the 95% confidence interval of maximum 7.4%-8.6%). This refers to the prevalence of health-related limitations in everyday activities (Global Activity Limitation Indicator, GALI), the most critical variable in the survey [20].

Table 1 provides information on the sample size achieved in the countries participating in EHIS 2. It also depicts both the reached effective sample size and the minimum effective sample size. The effective sample size is the size required if the survey was based on simple random sampling. The reached effective sample size was derived by dividing the reached sample size to the design effect provided for the GALI variable in national quality reports. The design effect describes the degree to which clustering and weighting can account for the increase of variance in complex survey designs. The ratio of the reached effective sample size to the minimum effective sample size indicates whether individual countries reached their target sample size. A value of at least 1 means the target sample size was achieved. However, as not all countries reported the design effect for the GALI variable, the corresponding data is not available for every country.

Nearly all the countries reached or even surpassed their specified effective sample size. Only a few countries failed to achieve the specified sample size despite high levels of participation. On the one hand, the design effect played a role as it affects the calculation. On the other hand, non-response rates during sampling may have been underestimated [15]. Failure to reach the specified effective sample size can lead to less precise prevalence estimators and thus to the non-detection of existing disparities in prevalences. Overall, 304,000 surveys were conducted in EU Member States, making EHIS 2 the largest health interview survey in the EU to date.

The target population in Germany is the German-speaking population aged 15 and above living in private households and registered with their primary residence in population registers [14]. A two-stage cluster sample was drawn. In the initial selection stage of the sampling procedure, the GESIS - Leibniz Institute for the Social Sciences selected 301 sample points at random from the total number of German municipalities (n=11,339) [14]. These represent the various sizes of municipalities and regions in Germany. The classification was based on the BIK classification, a regional classification system for Germany [23]. All federal states were taken into account. Less populous federal states were oversampled with a minimum of twelve sample points. In the second sampling stage, individuals with permanent residence in the sampled communities were drawn from the local population registers for each sample point. This drawing was stratified by age group (15 to 24, 25 to 34, 35 to 44, 45 to 54, 55 to 64, 65 to 74, 75 to 84, and older than 85), using a random statistical procedure (unrestricted random sampling) [14].

### 2.3 Study implementation

EU Member States were free to decide on the survey modes or combination of survey modes they used to implement the EHIS survey [20, 24]. In 16 out of 30 participating countries, the EHIS 2 survey was conducted using a single-mode design, i.e. using only one survey instrument; in the majority of cases, this took the form of face-to-face interviews, although telephone interviews and paper questionnaires were also used [15]. Fourteen countries used a mixed-mode design, i.e. a combination of several survey modes, for example combinations of self-administered paper-based and online questionnaires, face-to-face interviews plus supplementary self-administered questionnaires or telephone interviews followed up with paper questionnaires [15].

Article 4.3 of the implementation regulation specifies that data collection for EHIS 2 should take at least three months, with at least one month being in Autumn (September to November) [21]. The survey period was greater than three months in most countries [15], with the exception of Denmark, Italy, Lithuania, Hungary and Romania, where the EHIS 2 was completed within three months. The average survey period was eight months. Austria reported the longest survey period of 21 months, followed by Ireland with 19 months [15].

All Member States employing interviewers to implement EHIS provided courses and training sessions in the run-up to the survey. Generally, these involved detailed information about the survey, the questionnaire content, how to handle questions from participants, as well as the technical aspects of completing a questionnaire, for example during computer-assisted telephone interviews (CATI) or computer-assisted personal interviews (CAPI). The number of interviews per interviewer varied greatly between countries (15:1 in Austria and 248:1 in Cyprus). Denmark, Germany, Luxemburg and Finland relied exclusively on self-administered questionnaires and did not employ any interviewers [15].

In all EU Member States, initial contact with the selected participants was made via written invitation, with the exception of Ireland, where first contact was made via home visits. Further contact (e.g. reminders) was either made in writing, by telephone or in person. The number of such attempts to make contact and the method used also varied greatly between countries. Seven participating countries provided incentives to boost people’s willingness to participate in the study. These included shopping vouchers, payments in cash, shopping trolley tokens, reflective bands, keyrings and pens [15].

The duration of the interviews varied widely across Member States, depending on the survey mode and the form of implementation, for example as a stand-alone version of EHIS (leading to a shorter interview duration) or as part of a larger national health survey. On average, face-to-face interviews took between 20 and 47 minutes, while telephone interviews took between 20 and 65 minutes. In some participating EU countries, the duration of interviews was shortened by using surveys such as the European Labour Force Survey (EU-LFS) to supplement certain variables, e.g. for sociodemographics [15].

The use of proxy interviews, i.e. interviews in which third persons are asked about the actual target person was practiced differently in the participating countries. In twelve of the 30 EHIS 2 survey countries, proxy interviews were generally not permitted. These countries included Germany. In the remaining 18 countries, the proportion of proxy interviews varied between 13.4% in Belgium and 0.5% in Austria [15].

In Germany, EHIS was implemented using a sequential mixed-mode design, which means that the people invited to take part in the study could do so either online or in writing, options which were offered in chronological succession [14]. An initial letter sent by post invited potential participants to take part in the study online. People who did not participate online within four weeks of receiving the initial letter, or who did not explicitly state they did not wish to participate, were then sent the paper questionnaire via post. People who had still not responded after another three weeks were sent a reminder letter in the post.

The EHIS survey was conducted in Germany between November 2014 and July 2015, i.e. over a nine-month period. Incentives were provided to increase people’s willingness to participate. Participants aged 15 to 34 received a 10-Euro shopping voucher after completing the interview, and for participants 35 years and older, 400 shopping vouchers were raffled off to the value of 50 Euros each [14].

### 2.4 Survey instruments

The EHIS 2 questionnaire is comprised of four modules on health status, provision of healthcare, health determinants and sociodemographics [20]. Nearly all the EU Member States followed the suggested sequence of these question modules, with the exception of Belgium, Greece, Estonia, France, Italy, the Netherlands and Norway, where the sequence was modified [15]. Translation of the English language model questionnaire into the target languages of the respective EU Member States was based in nearly all the countries on the standardised translation protocol recommended by Eurostat[20]. Belgium, Spain, France, Lithuania, the Netherlands, Iceland and Norway were exceptions in this regard. In Spain, for example, a private translation company translated the questionnaire into the official regional languages (Catalan, Galician and Basque). EHIS was surveyed in a total of 27 languages. In 14 countries, the survey instrument was used in more than one language (e.g. in Luxemburg, where the survey was conducted in German, French, Portuguese and English) [15]. The EHIS manual contains the English language model questionnaire [20].

Germany and Austria jointly developed a German translation of the model questionnaire, which was then slightly modified by each country to reflect their use of different terms. The German GEDA 2014/2015-EHIS questionnaire has already been published and contains all of the EHIS 2 questions translated into German, as well as additional questions only surveyed in Germany [25].

### 2.5 Quality assurance, data management and data use

Article 6 of Commission Regulation (EU) No 141/2013 determines that EU Member States shall submit the finalised, validated and weighted microdata (as well as quality-related reference metadata) in accordance with an exchange standard specified by Eurostat using the Single Entry Point services [21]. Data preparation and quality assurance follow Eurostat‘s validation rules, which contain regulations on filter and value range checks, as well as plausibility checks [26]. A specially developed software programme was used to test whether the national data sets had been correctly adjusted. After receiving and testing all the data sets from the EU Member States, Eurostat compiled the complete EHIS 2 data set according to prescribed regulations on data protection and anonymization [27]. This data set can be applied for on the Eurostat website and may be used for scientific purposes only. Circulation and use of confidential EHIS data is regulated by Commission Regulation (EU) No 557/2013 [24]. According to this regulation, EHIS microdata may be used, not only for the statistical purposes of the European Statistical System (ESS) but also by research institutes for clearly defined scientific research purposes. This regulation, therefore, permitted the use of EHIS data in the analyses presented in this issue. Applying to access this data is a two-step process. Firstly, the research organisation must apply for the status of a recognised research institution. Secondly, the microdata file can be requested upon submission of a description of the research project [16]. The EU Member States own their data, and may veto attempts to access their country’s data set for a specific research project.

## 3. Response

All Member States went to considerable effort to achieve high response rates. As described above, up to five contact attempts were made in an effort to reach as many people as possible and get them to take part. Despite this, response rates in different Member States varied considerably. Denmark, Germany, Luxemburg, Austria and Finland, for example, reported response rates of less than 50%, whereas Cyprus and Portugal achieved over 90% [15]. There are a number of reasons for these differences. Even during the sampling process there were differences between the countries that could potentially affect the response. In the Czech Republic, for example, EHIS 2 was conducted as a follow-up survey to the EU-LFS. These participants had already agreed to further participation, and this had a positive effect on EHIS 2 response rates [15]. Disparities in response rates can also be traced to different ways in which proxy interviews were handled. As described above, some countries permitted the inclusion of proxy interviews, which had a positive effect on response rates (but a negative effect on quality), while other countries did not. Countries that relied exclusively on self-administered forms of data collection (such as Denmark, Germany, Luxemburg or Finland) generally registered the lowest response rates. In some countries, low response rates were primarily recorded for particular groups of participants, for example, elderly people in Austria, adolescents and men in Finland and Sweden, and younger people in the Czech Republic [15].

At 27.5%, the response rate in Germany was low. Over the past few years, a decline in response rates for health surveys has been observed in many European countries [28, 29]. However, a low response rate does not necessarily mean that a specific sample has a low level of representativeness. A comparison between the sampling distribution in GEDA 2014/2015-EHIS and the German population structure from 2014 shows that the GEDA 2014/2015-EHIS sample is highly representative and that the weighting adjustments were small. This indicates the sample’s high level of representativeness [14].

Significant disparities between Member States were also observed in relation to the non-response to certain questionnaire items (item non-response). Data on household income, in particular, was viewed as problematic and particularly sensitive. This was also the case with variables related to physical activity, alcohol consumption, mental health, inpatient and outpatient care, chronic diseases and preventive measures. In some instances, these questions were only answered by a small proportion of interviewees [15].

## 4. Weighting

Weighting factors were calculated individually by each Member State. Weighting was guided by the following objectives: to reduce non-response bias (i.e. a systematic distortion of the sample by non-participation), to reflect the sample design, and to adjust the sample to reflect specified population figures. Weighting ensures adequate consideration of the specific makeup of a country’s population. Eurostat’s guidelines had a minimum requirement that sex distribution and age distribution (in ten-year age groups) be adjusted to the target population [20]. Sample weights indicate the number of people represented by a participant in the target population. Weighting, therefore, ensures that each EU Member State is considered in proportion to its target population.

Due to the two-stage sample design, EHIS 2 weighting for international analyses in Germany consists of design and adjustment weighting [14]. Design weights correspond to the inverse of the selection probability of a participant in the sample point, multiplied by sample point selection probability (as of 31 December 2011, the date sample points were selected). The adjustment weight, adjusts the sample to the distribution of certain population characteristics. The population distribution is based on Federal Statistical Office data (federal state, age and sex as of 31 December 2014) [30]. The characteristics adjusted were age, sex, federal state and the settlement structure of district types as defined by the Federal Institute for Research on Building, Urban Affairs and Spatial Development (BBSR). The German sample shows an effectivity of 83.6% [31], which means that weighting increases the variance estimates by a factor of 1.196 (1/0,836) compared to the unweighted sample.

## 5. Discussion

Following the mandatory participation in European health surveys for all EU Member States, data on health, on the provision of healthcare, health determinants and the socioeconomic situation of the population aged 15 and above will be surveyed regularly every six years from the date of EHIS 2. Indicators and instruments for EHIS 2 were selected in an extensive evaluation and consensus process between the European countries. Eurostat provided a manual containing recommendations and guidelines on survey planning and implementation, as well as a model questionnaire, thereby ensuring a broadly comparable implementation of the survey across the EU Member States [20]. This makes the collected data suitable for both national analyses and European comparisons. For the first time, the standardisation of survey instruments in EHIS 2 allows for a direct comparison of prevalences across European countries for many indicators, in particular those relating to state of health and health determinants [10]. This provides opportunities for European comparisons that go beyond national health reporting. Overall, EHIS has thus established a basis for health monitoring with standardised core indicators at the European level. Continued use of the developed survey instruments should provide in particular highly significant comparative analysis of trends overtime. These analyses are also a valuable addition to national health reporting.

A comparative interpretation of the results from European countries needs to take into account that this data from EU Member States – within the framework set out in the Eurostat guidelines – has been collected using varying survey methods and sample designs. Depending on the indicator, the selected survey methods can influence results to a greater or lesser extent. Thus, questions on the utilisation of health services are less likely to be affected by trends, while distortions are more likely in areas such as health behaviour or chronic morbidity [32].

As well as taking indicator-related limitations into account, classification of results should observe country-specific differences including socioeconomic or cultural factors. Particularly in regard to the provision of healthcare, an evaluation of results is only possible if the strongly differing structures and care services within the healthcare systems of Europe are taken into consideration [33].

The European comparisons presented in this issue of the Journal of Health Monitoring are based on the population 15 years and older as specified for all EU Member States in EHIS 2. A comparison of prevalences with articles for Germany published to date, using GEDA 2014/2015-EHIS data needs to consider that national analyses only include the population 18 years and above and use a different weighting factor. The national weighting factor also adjusts for levels of education [14]. This means that GEDA 2014/2015-EHIS results in national analyses may differ from European analyses.

## Conclusion and outlook

EHIS data are collected and harmonised in EU Member States and are highly comparable, thus constituting an important information basis for European health policy and reporting.

In the articles in this issue of the Journal of Health Monitoring, EHIS 2 data applied for at Eurostat is evaluated in regard to educational differences in the prevalence of behavioural risk factors, partnership, parenthood, employment and self-rated health, depressive symptoms and limitations in activities of daily living.

Pursuant to Commission Implementation Regulation (EU) No 2018/255, the third European EHIS wave is scheduled for 2019 [34]. In Germany, the Robert Koch Institute implements EHIS 3 as part of the GEDA study. Data collection for GEDA 2019-EHIS began in April 2019. The first results, initially at a national level, can be expected in 2021.

## Key statements

- For the EHIS, EU Member States collect data every six years on the health, provision of healthcare, health determinants and socioeconomic situation of the population aged 15 and over.
- Indicators and instruments for the EHIS 2 were selected during an extensive evaluation and consensus process conducted by the European countries.
- EHIS is a population-based, cross-sectional survey based on the self-reporting of participants.
- EHIS 2 data were collected in the 28 EU Member States between 2013 and 2015.
- EHIS data are highly comparable and form an important information base for European health policy and health reporting.

## Figures and Tables

**Table 1:** Sample sizes in EHIS 2 participant countries Source: EHIS 2 quality report [15]

Participating country*	Reached sample size	Reached effective sample size	Minimum effective sample size	Ratio^1^
Austria	15,771	10,729	6,050	1.77
Belgium	9,113	4,297	6,500	0.66
Bulgaria	6,410	5,008	5,920	0.85
Croatia	5,446	–	–	–
Cyprus	4,958	4,948	4,095	1.21
Czech Republic	6,737	6,478	6,510	1.00
Denmark	5,811	–	5,350	–
Estonia	5,452	–	4,270	–
Finland	6,183	6,183	5,330	1.16
France	15,729	11,826	13,110	0.90
Germany	24,824	15,146	15,260	0.99
Greece	8,223	5,367	6,667	0.81
Hungary	5,826	6,905	6,410	1.08
Ireland	10,323	6,928	5,057	1.37
Italy	25,325	21,776	13,180	1.65
Latvia	7,077	9,870	4,555	2.17
Lithuania	5,205	6,426	4,850	1.32
Luxemburg	4,004	3,931	4,000	0.98
Malta	4,086	–	3,975	–
Netherlands	7,653	7,289	7,515	0.97
Poland	24,156	20,824	10,690	1.95
Portugal	18,204	–	6,515	–
Romania	16,605	–	8,420	–
Slovakia	5,490	5,719	5,370	1.06
Slovenia	6,262	4,673	4,486	1.04
Spain	22,842	14,929	11,620	1.28
Sweden	6,292	–	6,200	–
United Kingdom	20,161	14,130	13,085	1.08
Iceland^2^	4,001	–	3,940	–
Norway^2^	8,164	–	5,170	–

– missing data

* No Data available for Turkey

^1^ Ratio of the reached effective sample size to minimum effective sample size

^2^ No Member State of the European Union
